# The Association of Frailty With Long-Term Outcomes in Patients With Acute Respiratory Failure Treated With Noninvasive Ventilation

**DOI:** 10.7759/cureus.33143

**Published:** 2022-12-30

**Authors:** Mihaela Stefan, Aleezay Asghar, Meng-Shiou Shieh, Selma Demir-Yavuz, Jay S Steingrub

**Affiliations:** 1 Department of Medicine, University of Massachusetts (UMass) Chan Medical School - Baystate Medical Center, Springfield, USA; 2 Department of Internal Medicine, University of Massachusetts (UMass) Chan Medical School - Baystate Medical Center, Springfield, USA; 3 Department of Medicine, Griffin Hospital, Derby, USA; 4 Division of Pulmonary and Critical Care, Department of Medicine, University of Massachusetts (UMass) Chan Medical School - Baystate Medical Center, Springfield, USA

**Keywords:** critical illness, mortality, noninvasive ventilation, acute respiratory failure, frailty

## Abstract

The objective of this study was to investigate the prevalence and impact of frailty on mortality in patients with acute respiratory failure (ARF) treated with noninvasive ventilation (NIV). This was a single-center, prospective study of patients who developed ARF (irrespective of etiology) and were treated with NIV support. Frailty was assessed using the Clinical Frailty Scale (CFS). We modeled the relationship of CFS with one-year mortality using Cox proportional hazards regression, adjusting for other clinical and demographic characteristics. Of the 166 patients enrolled, 48% had moderate to severe frailty. These patients were more likely to be female (67% versus 33%) and on oxygen therapy at home (46% versus 28%). The median CFS score was 5 (interquartile range (IQR): 5-6). Moderate to severe frailty was associated with a 60% higher risk of one-year mortality (hazard ratio (HR): 1.63, 95% confidence interval (CI): 1.15-2.31). Frailty assessment may identify patients in need of ventilatory support who are at increased risk of mortality and may be an important factor to consider when discussing goals of care in this vulnerable population.

## Introduction

Noninvasive ventilation (NIV) is increasingly used to treat acute respiratory failure (ARF) and is effective in improving gas exchange and decreasing work of breathing [1-3]. The mortality of patients treated with NIV ranges between 20% and 52% at 30 days and 30% and 50% at one year [4,5]. Patients who fail NIV (i.e., intubation after an initial NIV trial) have higher mortality than patients who are intubated on presentation [6,7]. Among those who survive, almost 50% are readmitted within six months of discharge [8]. The decision to intubate and mechanically ventilate patients with progressive respiratory failure while on NIV remains a clinical dilemma. There is a critical need to risk stratify and identify patients with ARF being treated with NIV who are at high risk for death prior to making decisions to escalate therapy.

Frailty is a syndrome characterized by a generalized vulnerability to stressors resulting from an accumulation of physiologic deficits across multiple interrelated systems that result in poor functional status and disability [9]. Recent studies suggest that one in five critically ill patients younger than 50 and one in two patients older than 65 are frail [10]. Frailty has been identified as a risk factor for poor outcomes in adults with lung disease and critical illness, including younger individuals [11,12]. Frailty may have direct relevance to patients with ARF and may identify patients at greater risk for adverse outcomes and can facilitate shared decision-making with patients and surrogates regarding goals of care around the initiation of NIV support [13]. However, the extent to which the degree of baseline frailty is associated with survival remains unclear. The main objective of this study was to prospectively investigate the prevalence and impact of frailty on the risk of intubation and mortality in patients with ARF started on NIV therapy.

## Materials and methods

Design and setting

We performed a single-center, prospective study of consecutive patients who developed ARF (irrespective of etiology) and were treated with NIV support in a large, tertiary center in Western Massachusetts. We included patients aged 50 years or older, treated with NIV for ≥4 hours. We excluded patients with a Do Not Intubate (DNI) status, those on hospice or palliative care, or patients likely to die within 24 hours of admission based on the primary team’s clinical judgment. We also excluded patients unable to consent and did not have a surrogate to obtain consent from and those who did not speak English.

Key variable

Frailty was assessed using the Clinical Frailty Scale (CFS), which is an interview-based, validated, 9-point scale assessment that classifies patients along the continuum from very fit to severely frail [12]. Patients were asked to answer questions based on their state within two weeks prior to admission or when they were in their usual state of health such that it will reflect their baseline frailty. The next of kin were interviewed in lieu of patients who were unable to answer these questions (due to cognitive impairment, delirium, unresponsiveness, etc.). Two research assistants (RAs) interviewed patients and/or caregivers and determined the CFS. We considered patients to be frail if they had a score of 6 or greater (which represents moderate, severe, or very severe frailty). Grip strength was measured by means of a calibrated digital dynamometer (Jamar Hydraulic Hand Dynamometer, JLW Instruments, Chicago IL, USA). Three readings were taken and averaged to get the mean grip strength for each patient.

Outcomes

Our primary outcome was to assess one-year mortality in patients with frailty requiring NIV support. Secondary outcomes assessed NIV failure, mortality during hospitalization, length of stay in the hospital, and readmission at 30 days, six months, and 12 months. Mortality at one year was obtained from the National Death Index (NDI), which is a centralized national database of death record information on file in state vital statistics offices.

Variables collected included demographics, level of education, body mass index (BMI), the use of oxygen therapy at home, prehospital residence, principal diagnosis responsible for ARF, type of NIV, location of the initiation of NIV, number of days on NIV, and any end-of-life (EOL) initiatives (such as palliative care consult, goals of care discussion, change in code status, and decision to pursue hospice care). We also included comorbid conditions as per the Elixhauser Comorbidity Index [14].

Statistical analysis

Descriptive statistics according to frailty status were tabulated, and univariable comparisons of means, medians, and proportions were performed. Estimates are reported with 95% confidence intervals (CIs).

We modeled the relationship of frailty with death using Cox proportional hazards regression and included clinical and demographic characteristics as covariates to adjust for confounding. We included the CFS score (continuous) as the exposure in the model. A p-value of less than 0.05 was considered statistically significant for all comparisons.

The study was approved by the Institutional Review Board of the University of Massachusetts - Baystate (reference number: BH-17-124).

## Results

A total of 166 patients were enrolled with a median age of 69 (interquartile range (IQR): 61-76). Of the studied population, 49% were females, 86% were white, and 78% were living at home prior to admission. The most common comorbidities were hypertension (86%), diabetes mellitus (52%), renal disease (40%), and obesity (40%). The median Elixhauser Comorbidity Index [14] was 15 (IQR: 9-20), and the median grip strength was 43.7 pounds (IQR: 28.1-60.3). Of the patients, 40% had one or more activities of daily living (ADL) dependencies [15] and 60% had one or more instrumental activities of daily living (IADL) dependencies [16]. The median albumin value was 3.6 mg/dL (IQR: 3-4), and the median BMI was 32 (IQR: 25-40). The most common principal diagnoses responsible for ARF were congestive heart failure (CHF) exacerbation (34%), chronic obstructive pulmonary disease (COPD) (31%), and pneumonia (10%). NIV was started in the emergency department in most cases (79%). Of all patients, 11% were intubated and 20% were treated in the intensive care unit (ICU). Only 13% had EOL to variable degrees. Of these, palliative care was involved in 64% of these cases, and only 36% opted for hospice. The median length of stay in the hospital was eight days (IQR: 5-13); 58.4% of all patients were discharged to home and 34.3% to a nursing home. Additionally, 59% were readmitted one year post-discharge. The mortality rate in the hospital and at one year was 5% and 29%, respectively. The median CFS score was 5 (IQR: 5-6). The characteristics of patients in each group are illustrated in Table 1.

**Table 1:** Characteristics of the study population **patients who opted for hospice as a percentage of those who had EOL initiated ARF: acute respiratory failure; BMI: body mass index; CFS: Clinical Frailty Scale; CHF: congestive heart failure; COPD: chronic obstructive pulmonary disease; DM: diabetes mellitus; ED: emergency department; EOL: end of life; HTN: hypertension; ICU: intensive care unit; IQR: interquartile range; NIV: noninvasive ventilation; PHTN: pulmonary hypertension; PVD: peripheral vascular disease Values with an asterisk (*) were statistically significant with a p-value < 0.05.

Patient characteristics	All patients (number (%))	No-mild frailty (number (%))	Moderate-severe frailty (number (%))
	166	87 (52.4)	79 (47.6)
CFS score (median, IQR)	5 (5-6)	5 (4-5)	6 (6-6)
Age (median, IQR)	69 (61-76)	69 (59-76)	69 (62-78)
Gender (female) (number (%))*	82 (49.4)	29 (33.3)	53 (67.1)
Race (number (%))			
African American	14 (8.4)	6 (6.9)	8 (10.1)
Hispanic	10 (6.0)	3 (3.4)	7 (8.9)
Unknown	1 (0.6)	0 (0)	1(1.3)
White	142 (85.5)	78 (89.7)	64 (81.0)
Marital status (number (%))*			
Married	66 (39.8)	44 (50.6)	22 (27.8)
Other	100 (60.2)	43 (49.4)	57 (72.2)
Elixhauser comorbidities (number (%))			
CHF	120 (72.3)	62 (71.3)	58 (73.4)
Arrhythmia	94 (56.6)	52 (59.8)	42 (53.2)
Valvular disease	43 (25.9)	24 (27.6)	19 (24.1)
PHTN	32 (19.3)	17 (19.5)	15 (19.0)
PVD	29 (17.5)	17 (19.5)	12 (15.2)
HTN	142 (85.5)	72 (82.8)	70 (88.6)
Pulmonary	105 (63.3)	50 (57.5)	55 (69.6)
DM	23 (13.9)	14 (16.1)	9 (11.4)
DM with complications	63 (38.0)	29 (33.3)	34 (43.0)
Hypothyroidism*	41 (24.7)	15 (17.2)	26 (32.9)
Renal	66 (39.8)	30 (34.5)	36 (45.6)
Lymphoma	1 (0.6)	0 (0)	1 (1.3)
Metastases	3 (1.8)	2 (2.3)	1 (1.3)
Tumor	4 (2.4)	4 (4.6)	0 (0)
Obesity*	66 (39.8)	25 (28.7)	41 (51.9)
Weight loss	10 (6.0)	8 (9.2)	2 (2.5)
Blood loss	2 (1.2)	1 (1.1)	1 (1.3)
Anemia	15 (9.0)	8 (9.2)	7 (8.9)
Alcohol	4 (2.4)	2 (2.3)	2 (2.5)
Drugs	13 (7.8)	8 (9.2)	5 (6.3)
Psychoses	2 (1.2)	1 (1.1)	1 (1.3)
Depression	49 (29.5)	24 (27.6)	25 (31.6)
Elixhauser Comorbidity Index (IQR)	15 (9-20)	3.7 (3.1-4.1)	12 (8-20)
Albumin (median (IQR))	3.6 (3.0-4.0)	3.7 (3.1-4.1)	3.5 (3.0-3.9)
BMI (median (IQR))*	31.8 (24.8-40.2)	30.5 (23.9-35.5)	35.3 (26.7-42.6)
On home oxygen (number (%))*	60 (36.1)	24 (27.6)	36 (45.6)
Grip strength (lb) (median (IQR))*	43.7 (28.2-60.3)	52.7 (37.9-67.5)	35.7 (24.5-47.2)
Number of hospital admissions in previous year (median (IQR))*	1 (0-3)	1 (0-2)	2 (1-4)
Admitted from (number (%))			
Home	130 (78.3)	68 (78.2)	62 (78.5)
Nursing home	21 (12.7)	9 (10.3)	12 (15.2)
Other clinical facility	15 (9.0)	10 (11.5)	5 (6.3)
Principal diagnosis of ARF (number (%))			
COPD	51 (30.7)	24 (27.6)	27 (34.2)
CHF	57 (34.3)	29 (33.3)	28 (35.4)
Asthma	5 (3.0)	1 (1.1)	4 (5.1)
Pneumonia	16 (9.6)	11 (12.6)	5 (6.3)
Sepsis	4 (2.4)	2 (2.3)	2 (2.5)
Other cardiovascular	13 (7.8)	8 (9.2)	5 (6.3)
Other pulmonary	13 (7.8)	10 (11.5)	3 (3.8)
Other	7 (4.2)	2 (2.3)	5 (6.3)
Location of the initiation of NIV (number (%))			
ED	131 (78.9)	68 (78.2)	63 (79.7)
Ward	19 (11.4)	9 (10.3)	10 (12.7)
ICU	16 (9.6)	10 (11.5)	6 (7.6)
Intubation (number (%))	18 (10.8)	11 (12.6)	7 (8.9)
Admission to ICU (number (%))	33 (19.9)	22 (25.3)	11 (13.9)
Length of hospital stay (days) (median (IQR))	8 (5-13)	8 (5-14)	7 (5-12)
Any EOL (number (%))			
No	144 (86.7)	78 (89.7)	66 (83.5)
Yes	22 (13.3)	9 (10.3)	13 (16.5)
Palliative care consult (number (%)**)	14 (63.6)	5 (55.6)	9 (69.2)
Hospice (number (%)**)	8 (36.4)	3 (33.3)	5 (38.5)
Discharge location (number (%))			
Home	43 (25.9)	27 (31.0)	16 (20.3)
Home with health services	54 (32.5)	25 (28.7)	29 (36.7)
Nursing home	57 (34.3)	30 (34.5)	27 (34.2)
Death	9 (5.4)	2 (2.3)	7 (8.9)
Another hospital	3 (1.8)	3 (3.4)	0 (0)
Discharged to hospice	12 (7.2)	7 (8.0)	5 (6.3)
Readmission rates among survivors (number (%))			
30 days post-hospitalization	36 (22.9)	15 (17.6)	21 (29.2)
180 days post-hospitalization	80 (51.0)	40 (47.1)	40 (55.6)
365 days post-hospitalization	92 (58.6)	47 (55.3)	45 (62.5)
Mortality at one year (number (%))	45 (28.7)	21 (24.7)	24 (33.3)

Comparison between frail and non-frail patients

**Figure 1:**
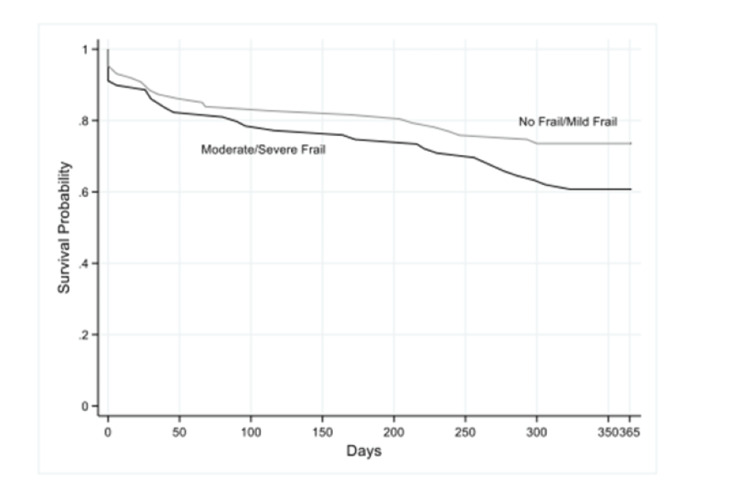
Probability of survival in relation to frailty over one year

Overall, 48% of all patients had moderate to severe frailty and were more likely to be female (67% versus 33%), unmarried (72% versus 49%), and on oxygen therapy at home (46% versus 28%). This subset was also noted to have lower grip strength (35.7 (IQR: 24.5-47.2) versus 52.7 (IQR: 37.9-67.5)) and was more likely to have one or more ADL and IADL dependency (76% versus 29% and 64% versus 31%, respectively). A revision in code status to Do Not Resuscitate (DNR) or Do Not Intubate (DNI) occurred in 13% of the moderate to severe frailty group compared to 4% in those with no to mild frailty group. In terms of outcomes, 9% of all patients in the moderate to severe frailty group died during their hospitalizations versus 2% of those in the no to mild frailty group. One-year mortality and 30-day readmission rates were also higher in the frailty group (33% versus 25% and 29% versus 18%, respectively), but the frailty group had a lower NIV failure rate. In the Cox proportional analysis adjusted for demographics and clinical characteristics, frailty was associated with mortality at one year (hazard ratio (HR): 1.63; 95% CI: 1.15-2.31) as demonstrated in Figure 1.

## Discussion

In this study of 166 patients hospitalized with ARF and treated with NIV, frailty was identified in approximately 78% of patients, with 48% having moderate to severe frailty. Frailty was associated with an increased risk of in-hospital and long-term mortality, and readmission rate. Our results extend the findings from other studies that assessed the association between frailty and outcomes in critically ill patients [10,11]. Although frail patients had higher rates of adverse outcomes, it is worth noting that one-year mortality and readmissions were high for all patients.

NIV is the first-step treatment strategy in patients with ARF secondary to COPD or CHF, but it is also widely used in conditions for which the evidence remains weak [17]. Prior studies have shown that NIV is used preferentially in elderly patients [18]. More recently, there has been increased recognition of the impact of frailty on the outcomes of critically ill patients. A recent study by Hendin et al. concluded that frailty was associated with significantly fewer days alive at home within a year of a critical care admission due to long-term care admissions, hospital readmissions, and increased mortality [19].

A meta-analysis of 11 studies evaluating elderly patients admitted to ICU found that frailty was associated with an increased risk of in-hospital mortality (RR: 1.73; 95% CI: 1.55-1.93) and long-term mortality (six studies) (RR: 1.86; 95% CI: 1.44-2.42) [10]. Our results are very similar to those presented in this meta-analysis, with an HR of 1.63 (95% CI: 1.15-2.31). In our study, mortality at one year was 29%, which is lower than the estimate reported in a large study of Medicare patients by Lindenauer et al. (42%), but our study excluded patients with a DNR status at the time of NIV initiation and included patients 50 years or older [8].

Frailty assessment tools can be used at the bedside to help clinicians, patients, and families in shared decision-making regarding aggressive end-of-life treatments. Although there is no gold standard test for determining frailty, several validated, user-friendly tools that assess frailty such as CFS are available and can reliably predict survival outcomes in critically ill patients [20]. However, the use of frailty assessment tools in the hospital and particularly in the critical care setting has been limited [21], and there is no validated model to our knowledge that uses this information to predict clinical outcomes in this specific population.

In our study, almost half of the patients had moderate to severe frailty and only 22% were non-frail; these results are comparable with those from a recent study by Kara et al. [13] but higher than those reported in prior studies that included ICU patients and did not focus on patients with ARF using NIV [22]. Paradoxically, the NIV failure rate in the severely frail patients was lower than that observed in the non-frail to mildly frail patients in our study, possibly reflecting the higher rate of revision in the code status to a DNR/DNI status in these patients, which precluded escalation to intubation. It is also possible that patient selection for NIV therapy reflects local institutional practice. Elderly frail patients are more likely to have delirium and dementia with altered mental status, and NIV is less likely to be used in these individuals in our hospital due to the risk of aspiration and intolerance/lack of cooperation.

Although more than 60% of all patients had ADL and IADL dependency and more than half had severe frailty, only 8% of all patients had a palliative care consult during hospitalization. Involving the palliative care teams earlier during the admission of critically ill patients could better clarify their goals of care and support families in making decisions respecting the patients’ wishes.

Limitations

In addition to what is discussed above, our study’s limitations include that it was a single-center study, and the results could be influenced by the practices in this institution. Given that this was an observational study, our study may not have accounted for unmeasured confounders, as is the case with any observational analysis. Additionally, we only recruited English-speaking patients, so it is likely that certain ethnicities were underrepresented in our study.

## Conclusions

Our study showed that frailty is highly prevalent in elderly patients hospitalized with ARF and treated with NIV and is a predictor of poorer outcomes, with an approximately 60% higher risk of one-year mortality as well as an increased risk of in-hospital mortality and readmission rate. Frailty assessment is an important factor to consider when discussing the goals of care with these vulnerable patients. Our study highlights that the development of a validated predictive model using CFS may be beneficial in predicting outcomes among frail patients with ARF requiring NIV.
